# Unearthing the Alleviatory Mechanisms of Brassinolide in Cold Stress in Rice

**DOI:** 10.3390/life12060833

**Published:** 2022-06-02

**Authors:** Qingshan Xu, Qianqian Wei, Yali Kong, Lianfeng Zhu, Wenhao Tian, Jing Huang, Lin Pan, Qianyu Jin, Junhua Zhang, Chunquan Zhu

**Affiliations:** 1 State Key Laboratory of Rice Biology, China National Rice Research Institute, Hangzhou 310006, China; xuqingshan1998@126.com (Q.X.); m15955803543@163.com (Q.W.); kongyali@caas.cn (Y.K.); zlfnj@163.com (L.Z.); tianwenhao@caas.cn (W.T.); hj15163859612@163.com (J.H.); panlin0610@126.com (L.P.); jinqianyu@caas.cn (Q.J.); 2 School of Resources and Environmental Engineering, Anhui University, Hefei 230039, China; 3 College of Life Sciences and Technology, Mudanjiang Normal University, Mudanjiang 230039, China

**Keywords:** rice, brassinolide, cold stress, osmotic substance, antioxidant enzymes, gene regulation

## Abstract

Cold stress inhibits rice germination and seedling growth. Brassinolide (BR) plays key roles in plant growth, development, and stress responses. In this study, we explored the underlying mechanisms whereby BR helps alleviate cold stress in rice seedlings. BR application to the growth medium significantly increased seed germination and seedling growth of the early rice cultivar “Zhongzao 39” after three days of cold treatment. Specifically, BR significantly increased soluble protein and soluble sugar contents after three days of cold treatment. Moreover, BR stimulated the activity of superoxide dismutase, catalase, peroxidase, and ascorbate peroxidase; thereby alleviating cold-induced damage and increasing glutathione content and the GSH/GSSG ratio while concomitantly reducing H_2_O_2_ content. BR upregulated the expression levels of cold-response-related genes, including *OsICE1*, *OsFer1*, *OsCOLD1*, *OsLti6a*, *OsSODB*, *OsMyb*, and *OsTERF2,* and downregulated that of *OsWRKY45*, overall alleviating cold stress symptoms. Thus, BR not only upregulated cellular osmotic content and the antioxidant enzyme system to maintain the physiological balance of reactive oxygen species under cold but, additionally, it regulated the expression of cold-response-related genes to alleviate cold stress symptoms. These results provide a theoretical basis for rice breeding for cold resistance using young seedlings.

## 1. Introduction

Rice (*Oryza sativa* L.) is a tropical plant that grows in high-temperature and humid environments. Therefore, cold stress inhibits rice germination and growth. For example, cold stress enhances seed respiration, which affects subsequent seedling growth [1]. Furthermore, during the seedling stage, cold stress inhibits chlorophyll synthesis and reduces photosynthetic rate, root vigor, and tillering and, consequently, panicle number, ultimately reducing grain weight and crop yield [2]. In addition, cold stress induces cotton rot infection in rice, which causes plant death [3]. Therefore, it is important to avoid cold stress or improve cold resistance during rice growth, especially during early seedling growth, to ensure high crop productivity.

Plants have evolved various strategies to adapt to cold stress, which induces an increase in the production of reactive oxygen species (ROS), especially hydrogen peroxide (H_2_O_2_), in plant cells. In turn, cold-induced increased H_2_O_2_ acts as a signal that stimulates the cellular ROS-scavenging system [4]. Particularly, the enzymatic scavenging system, which includes superoxide dismutase (SOD), peroxidase (POD), catalase (CAT), and ascorbate peroxidase (APX), forms the first line of ROS scavengers in plant tissues. Specifically, SOD catalyzes the conversion of oxygen free radicals (O_2_^·−^) to H_2_O_2_, while POD, CAT, and APX catalyze the conversion of H_2_O_2_ to H_2_O to alleviate ROS stress [5,6]. In maize leaves, SOD, POD, and APX activities reportedly increase significantly at 5 °C, resulting in enhanced plant resistance to cold stress [7]. Similarly, low temperatures reportedly enhance SOD, CAT, and APX activity levels in soybeans [8].

In addition to antioxidant enzyme activity, the non-enzymatic scavenging system, which includes vitamins A, C, E, ascorbic acid, and ascorbic acid sulfur-based compounds, is also involved in alleviating cold-induced ROS stress [9,10,11]. For example, cold treatment of spinach at 10 °C can significantly increase ascorbic acid content by 41%, thereby enhancing plant resistance to cold and improving the nutritional quality of spinach leaves [12]. Furthermore, plants can reduce the osmotic and freezing points of cells at low temperatures by accumulating osmotically active substances, such as proline and soluble sugars, to adapt to low ambient temperature [13].

In addition, genes associated with cold resistance have been identified in rice [14]. Specifically, the protein encoded by *COLD1* interacts with the G protein to activate Ca^2+^ channels, which in turn rapidly increase Ca^2+^ flow in rice root cells, thereby regulating rice tolerance to cold. Consistently, overexpression of *OsCOLD1* significantly increases cold tolerance in rice [15], and overexpression of *OsLti6a* and *OsLti6b* protects the integrity of the plasma membrane and alleviates cold stress symptoms [16]. Further, overexpression of *TERF2* not only increases the osmotic content and chlorophyll but reduces ROS and malondialdehyde contents as well, thereby reducing membrane electrolyte leakage and alleviating cold stress [17].

Improving cold resistance in rice is crucial for successful early development of rice seedlings grown at low temperatures [18]; several attempts have been made in this direction. For example, cold acclimatization as well as the application of salicylic acid and nitric oxide increase the survival and growth of seedlings under cold-stress conditions [19,20,21]. Similarly, the plant hormone brassinolide (BR) not only regulates plant growth and development [22,23,24,25] but also plant responses to cold stress [26,27]. Under normal conditions, BR improves plant growth by increasing photosynthetic efficiency [28]. Further, under cold-stress conditions, application of BR significantly decreases the number of free radicals by increasing the production of free radical scavengers, reducing membrane lipid peroxidation, and stabilizing cell membrane structure and function. Overall, these effects contribute to alleviating cold-stress symptoms in plants [29]. As BR is involved in plant cold resistance, we hypothesized that it is possible to improve early rice germination and growth under cold conditions by adding BR to the growth substrate while raising seedlings. The mechanism whereby BR alleviates cold-stress symptoms is also important for early rice production.

## 2. Materials and Methods

### 2.1. Rice Growth Conditions and Experimental Design

The rice material used for the experiments in this study was the indica-type, conventional, early rice variety “Zhongzao 39”, and the seedling growth substrate used was the fermentation substrate developed by the China National Rice Research Institute (Hangzhou, China).

The basic physical and chemical properties of the substrate were as follows: electrical conductivity, 3.12 ± 0.25 mS·cm^−1^; bulk density, 0.62 ± 0.03 g·cm^−3^; pH, 7.09 ± 0.0; and cation exchange capacity, 20.3 ± 2.02 cmol·kg^−1^. As for macronutrient contents, total N was 8.99 ± 0.84 g·kg^−1^, total P was 7.66 ± 0.24 g·kg^−1^, total K was 9.45 ± 0.08 g·kg^−1^, available N was 640.03 ± 10.11 mg·kg^−1^, available P was 285.16 ± 3.23 mg·kg^−1^, and available K was 2639.64 ± 14.73 mg·kg^−1^.

Germination percentage and seedling cold-tolerance experiments were performed to explore the role of BR in promoting germination of rice seeds and improving cold tolerance of rice seedlings, respectively. The specific experimental design was as follows.

#### 2.1.1. Determination of Germination Percentage

Rice seeds were washed thrice, and floating grains were removed immediately. After treatment with 2.6% NaClO for 30 min, seeds were rinsed with deionized water and soaked in clean water for 48 h and then washed thrice. Subsequently, 1000 seeds were sown in the growth substrate with or without 0.0001% 28-epihomobrassinolide (BR) (28-epihomobassinolide produced by Yunnan Yunda Technology Agrochemical Co., Ltd., Kunming, China, purity 90%) and subjected to low (15 °C day/10 °C night) or room temperature (30 °C day/20 °C night) in an incubator for three days. The seeds were then cultivated in the greenhouse of the China Rice Research Institute. The number of germinated seeds was recorded every day for seven days after cultivation, and the germination rate and germination potential were calculated according to the following formulae:

Germination rate (%) = (total germination after 7 d/number of seeds) × 100%.

Germination potential (%) = (number of germinated seeds on the third day/number of seeds) × 100%.

#### 2.1.2. Determination of Seedling Cold Tolerance

After soaking the rice seeds as mentioned above, 90 g of the seeds were sown in the substrate, with or without 0.0001% BR, on 7 September 2021, and the germination trays were placed in a greenhouse for seven days. The seedling trays were then placed in an artificial incubator for cold cultivation (15 °C, daytime/10 °C, night) for three days. Simultaneously, an ambient temperature (30 °C, daytime/20 °C, night) control was set. The different experimental treatments are shown in Table 1. Three replicates per treatment were included. “W + H” represents the seedling substrate without BR application or cold treatment (control); “B + H” represents the seedling substrate with BR application and without cold treatment, “W + L” represents the seedling substrate without BR application but with cold treatment, and “B + L” represents the seedling substrate with BR and cold treatment application. The BR concentration in the substrate was selected based on a preliminary experiment summarized in Supplementary Materials Table S1.

### 2.2. Determination of Seedling Growth Parameters

After the cold test, 20 seedlings were randomly selected from each seedling tray to measure plant height, leaf age, number of roots, and stem base width. Additionally, the dry weight of 100 plants was recorded.

### 2.3. Detection of Soluble Sugar and Soluble Protein

Fresh leaves were cut off, ground in liquid nitrogen, and 0.1 g was weighed to determine soluble sugar and soluble protein content. Soluble sugar content was determined by anthrone colorimetry. Under the action of concentrated sulfuric acid, sugar can undergo dehydration to generate uronic acid or hydroxymethyl furfural, both of which can be combined with anthrone to generate a blue-green uronic derivative. Within a certain range, the shade of the color is indicative of sugar content [30,31]. In turn, soluble protein yielded a cyan coloring after binding to Coomassie Brilliant blue G-250 in a dilute acid environment, and the absorbance was measured at 595 nm. Simultaneously, a standard protein concentration curve was prepared using 100 μg/mL bovine serum albumin solution, and the content of soluble protein in rice was calculated by substituting absorbance readings into the standard curve [32]. The procedure was as follows: 0.1 g of Coomassie Brilliant blue G-250 was weighed and dissolved in 50 mL of 90% ethanol, followed by the addition of 100 mL of 85% (*w*/*v*) phosphoric acid to obtain a final volume of 1 L to prepare the Coomassie Brilliant blue G-250 solution. Meanwhile, 0.1 g of liquid nitrogen-ground fresh plant sample was weighed, to which 1 mL distilled water was added and mixed thoroughly to extract the soluble protein, followed by centrifugation at 3000 rpm for 10 min. Subsequently, 0.2 mL of the supernatant was withdrawn, to which 1 mL of the Coomassie Brilliant blue G-250 solution was added and mixed thoroughly. After 2 min, colorimetric measurement was performed at 595 nm, and the protein content was estimated using the standard protein concentration curve.

### 2.4. Determination of H_2_O_2_ Content and Antioxidant Enzyme Activity

Fresh rice leaves were crushed under liquid nitrogen immediately after cutting, and the physiological parameters were determined on ice. Determination of H_2_O_2_ content was based on the formation of a yellow complex precipitate upon addition of titanium chloride, which was then dissolved in sulfuric acid [33]. The specific experimental procedure was as follows: 0.1 g of the leaf sample was weighed into a 2 mL centrifuge tube, and 1 mL of acetone solution was added. After shaking thoroughly for 15 s, the mixture was centrifuged at 5000 r/min for 10 min. Subsequently, 0.2 mL of the supernatant was collected and placed in a fresh test tube, to which 0.1 mL of 5% titanium sulfate and 0.2 mL of concentrated ammonia water were added, followed by centrifugation at 5000 r/min for 10 min. After formation of the precipitate, the excess supernatant was drawn out under a vacuum using a liquid transfer gun. Subsequently, 1 mL of 2 M sulfate acid was added to the precipitate, and the absorbance was determined at 410 nm after the precipitate was dissolved. Meanwhile, the absorbance of an H_2_O_2_ solution with a standard concentration gradient was determined under the same treatment conditions, and a linear curve of standard concentration gradient was obtained. Finally, H_2_O_2_ content in the rice samples was calculated. SOD activity was determined using the nitroblue tetrazolium (NBT) photoreduction method [34]. CAT activity was measured according to the consumption of H_2_O_2_ per unit of time [35]; POD activity was determined using the guaiacol method [36], and APX activity was determined by the reduction in ascorbic acid content [37].

### 2.5. Detection of AsA-GSH Circulating Substances

Ascorbic acid (AsA) content was determined using the fast blue salt B colorimetric method. Briefly, in an acetic acid solution, AsA reacts with fast blue salt B to form a yellow oxalazide-2-hydroxybutyrylactone derivative, and the absorbance is measured at the maximum absorption wavelength of 420 nm [38]. Dehydroascorbate (DHA) was reduced to generate AsA, and DHA content was calculated by measuring the AsA generation rate in the system. Glutathione (GSH) content was determined using the 2-nitrobenzoic acid (DTNB) colorimetric method. DTNB reacts with GSH to form a complex that has a characteristic absorption peak at 412 nm, and its absorbance is proportional to GSH content. Oxidized glutathione (GSSG) was determined using the 2-vinylpyridine (2-VP) method [39].

### 2.6. RNA Extraction and Gene Expression

Fresh rice leaves were ground with liquid nitrogen immediately after cutting, and total leaf RNA was extracted by adding TRizol. The content and purity of the total RNA were determined using NanoDrop, and the integrity of the extracted total RNA was determined by agarose gel electrophoresis. Total RNA was then reverse-transcribed into cDNA (PrimeScript Reverse Transcription Kit, TaKaRa, Shiga, Japan), and real-time qRT-PCR was performed using Sybgreen (TaKaRa, Japan). Primers for the selected genes and internal reference genes are shown in Supplementary Materials Table S2. *OsHistone* was used as the internal reference gene, and the treatment group without BR was used as the control under room-temperature conditions.

### 2.7. Statistical Analysis

Statistical analysis (one-way ANOVA) was performed on the experimental data using SAS9.2 software, and Tukey’s test was used for statistical differences. Different letters in the figures and tables indicate that the differences between treatment means were significant (*p* < 0.05).

## 3. Results

### 3.1. BR Improved Rice Germination Percentage under Cold-Stress Conditions

Cold stress significantly inhibited germination and germination potential in rice, reducing them from 90.33% to 87.66% and from 84.33% to 79.66%, respectively. However, the application of BR under cold-stress conditions significantly increased the rice germination percentage from 87.66% to 89.33% and seed germination potential from 79.66% to 83.66%. In addition, BR increased germination percentage and germination potential under normal temperature conditions (Table 2).

### 3.2. BR Improved Rice Seedling Growth under Cold-Stress Conditions

Compared to those with the normal temperature treatment, cold stress significantly reduced plant height, root number, leaf age, and shoot width. Application of BR effectively reversed cold-stress-induced rice growth inhibition, as it increased plant length by 3.97%, plant weight by 5.69%, root number by 14.43%, and shoot width by 1.74% (Table 3).

### 3.3. BR Increased Soluble Protein and Soluble Sugar Contents in Rice under Cold-Stress Conditions

**Figure 1:**
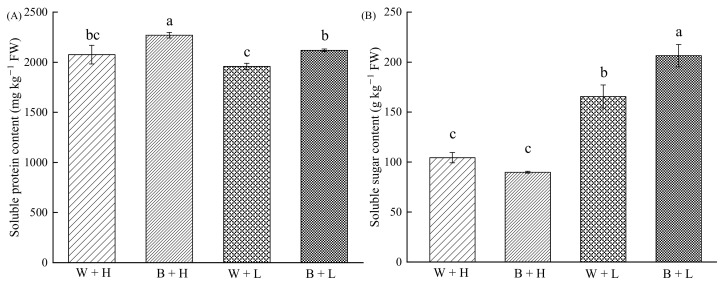
The effect of BR on soluble protein and soluble sugar contents in rice seedlings exposed to contrasting temperature regimes. Different letters in the figures indicate that the differences between treatment means were significant (*p* < 0.05). (**A**) Soluble protein content of rice seedlings under different treatments, (**B**) Soluble sugar content of rice seedlings under different treatments.

Compared to that with the ambient temperature treatment, cold stress significantly reduced soluble protein content of rice seedlings by 5.61%, while the application of BR increased it by 6.15% (Figure 1A). Conversely, cold stress significantly increased soluble sugar content in rice seedlings by 58.60%, and BR application further increased it by 24.70% (Figure 1B).

### 3.4. BR Regulated the Activity of Antioxidant Enzymes and Reduced the H_2_O_2_ Content under Cold Stress

Compared with those in ambient temperature, cold stress significantly inhibited SOD, POD, and APX activities by 9.73%, 14.35%, and 33.26%, respectively, whereas it increased CAT activity by 7.09% and H_2_O_2_ content by 4.70% (Table 4). In contrast, the application of BR reversed the inhibition of antioxidant enzyme activity; thus, POD, APX, CAT, and SOD activities increased by 10.70%, 23.67%, 21.39%, and 18.27%, respectively, while H_2_O_2_ content decreased by 11.34% (Table 4).

**Figure 2:**
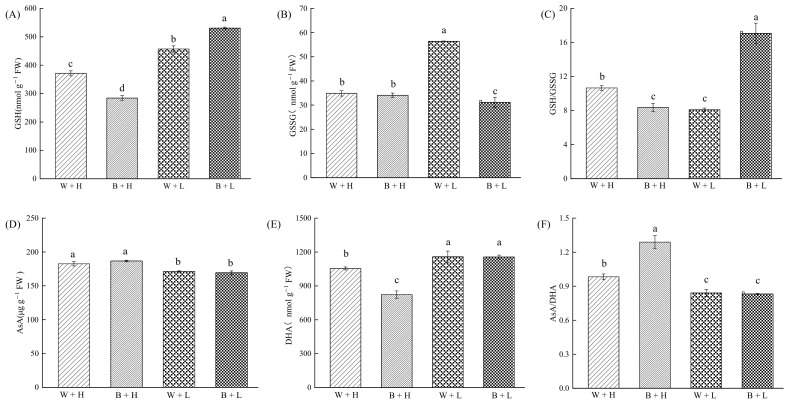
The effect of BR on the ascorbic acid-glutathione oxidative system in rice seedlings exposed to contrasting temperature regimes. Different letters in the figures indicate that the differences between treatment means were significant (*p* < 0.05). (**A**) GSH content of rice seedlings under different treatments, (**B**) GSSH content of rice seedlings under different treatments, (**C**) GSH/GSSH ratio of rice seedlings under different treatments, (**D**) AsA content of rice seedlings under different treatments, (**E**) DHA content of rice seedlings under different treatments, (**F**) AsA/DHA ratio of rice seedlings under different treatments.

Cold stress significantly increased GSH, GSSG, and DHA contents by 23.11%, 61.74%, and 9.77%, respectively, while reducing AsA content by 6.14%, the GSH/GSSG ratio by 23.91%, and the AsA/DHA ratio by 14.41%. The application of BR significantly increased GSH content by 16.14% (Figure 2A) but decreased GSSG content by 44.73% (Figure 2B), while it increased the GSH/GSSG ratio by 110.80% (Figure 2C). Meanwhile, there were no significant changes in AsA or DHA contents or the AsA/DHA ratio.

### 3.5. Relative Expression of Cold-Resistance Genes

**Figure 3:**
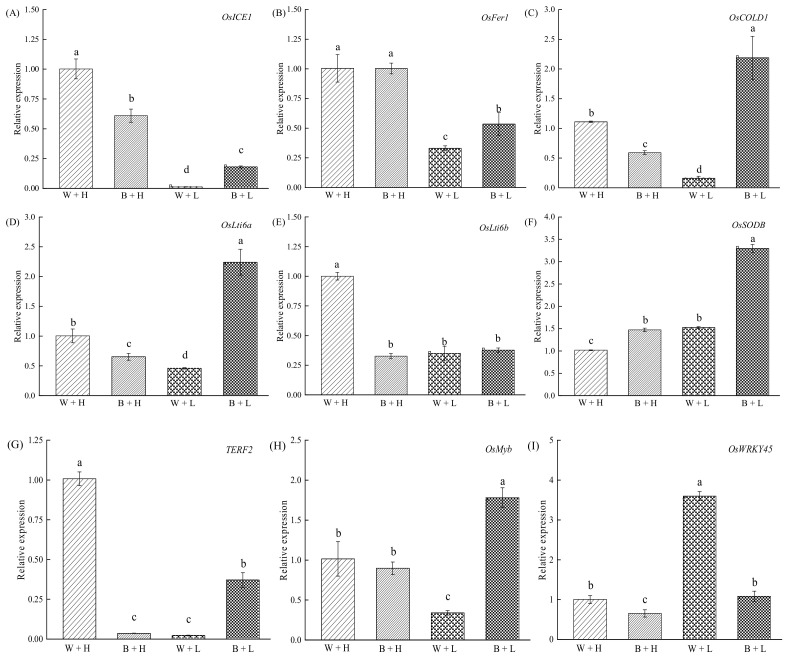
The effect of BR on relative expression of cold-resistance genes in rice seedlings exposed to contrasting temperature regimes. Different letters in the figures indicate that the differences between treatment means were significant (*p* < 0.05). (**A**) Relative expression of *OsICE1* in rice seedlings under different treatments, (**B**) Relative expression of *OsFer* in rice seedlings under different treatments, (**C**) Relative expression of *OsCOLD1* in rice seedlings under different treatments, (**D**) Relative expression of *OsLti6a* in rice seedlings under different treatments, (**E**) Relative expression of *OsLti6b* in rice seedlings under different treatments, (**F**) Relative expression of *OsSODB* in rice seedlings under different treatments, (**G**) Relative expression of *TERF2* in rice seedlings under different treatments, (**H**) Relative expression of *OsMyb* in rice seedlings under different treatments, (**I**) Relative expression of *OsWRKY45* in rice seedlings under different treatments.

In this study, cold stress significantly inhibited the expression of the cold-response genes *OsICE1*, *OsFer1*, *OsCOLD*, *OsLti6a*, *OsLti6b*, *OsMyb*, and TERF2 in rice, and increased the expression of *OsSODB*. In turn, the application of BR significantly enhanced the expression of *OsICE1*, *OsFer1*, *OsCOLD*, *OsLti6a*, *OsSODB*, *OsMyb*, and *TERF2* (Figure 3); however, the expression of *OsWRKY45* was reduced by BR compared with that in cold treatment (Figure 3).

## 4. Discussion

### 4.1. BR Improved Rice Germination and Growth under Cold-Stress Conditions

The promotion of plant growth and development and resistance to stress by addition of BR have been demonstrated in previous studies. For example, when *Medicago sativa* seeds were treated with 0.000001% BR (*w*/*w*), the germination percentage increased significantly, and the time required for plant germination was significantly reduced [40]. Further, the application of BR improved the germination rate of rice seeds by increasing free proline and soluble protein contents under salt stress, and the conclusion that BR treatment aids in overcoming salt-stress-induced inhibition of seed germination was also validated in Arabidopsis and *Brassica napus* [41]. In addition, Arabidopsis seedlings showed enhanced development of lateral roots under a 0.000005% BR (*w*/*w*) treatment, which caused an increase in auxin polar transport [42]. In this study, adding 0.0001% BR to the substrate matrix significantly increased seed germination percentage and germination potential in rice grown under cold-stress conditions, suggesting that BR also alleviated cold stress in rice during the germination stage. The higher BR concentration in the present study than in previous studies might have been due to the different plant species. In addition, rice growth parameters, including plant height, dry weight, and root number significantly increased after 3 days of cold treatment, suggesting that BR also increased the cold resistance of rice seedlings.

### 4.2. BR Regulated Osmotic Materials to Enhance Cold Resistance in Rice

Osmotic regulators, such as soluble sugars and soluble proteins, improve plant oxidative tolerance by directly participating in ROS quenching to maintain cell membrane integrity and reducing the freezing point for plants to adapt to cold stress [43]. Soluble sugars provide a large amount of carbon skeletons for normal metabolism, such as energy production, thereby enhancing cold tolerance in plants. Soluble proteins combine with water to maintain cell water content and thus reduce physiological water loss under cold-stress conditions [44]. In addition, soluble proteins improve plant cold tolerance by affecting gene expression [45]; however, low temperatures cause the decomposition of soluble proteins, resulting in a gradual decrease in soluble protein content [46]. Therefore, improving soluble sugar and soluble protein contents is important for enhancing cold resistance in plants. BR reportedly exhibits a significant effect on promoting soluble sugar and soluble protein contents of bitter gourd, wheat, and rice under cold stress [47,48]. In this study, the addition of BR under cold conditions significantly increased soluble sugar and soluble protein contents in rice seedlings, and thus, could improve cold resistance of rice seedlings.

### 4.3. BR Maintained ROS Balance to Alleviate Cold-Stress Symptoms in Rice

Numerous studies have shown that when stress disrupts cell homeostasis, the dynamic balance between production and removal of ROS, such as singlet oxygen, O_2_^·−^ and H_2_O_2_, is also disrupted, resulting in excessive ROS accumulation in cells [4,7,8]. The stress-induced burst of ROS in plant tissues causes oxidative damage to lipids, proteins, and nucleic acids [49]. Antioxidant enzyme systems are considered important plant defense systems under environmental stress. As a first line of defense, SOD can specifically convert O_2_^·−^ into H_2_O_2_ and O_2_. Subsequently, H_2_O_2_ is scavenged by APX, CAT, and POD activities [50]. Thus, BR-treated rice seedlings showed significantly increased CAT, SOD, and GR activities under salt stress [51]. BR exerts a protective effect by improving the antioxidant system against adverse reactions resulting from cold stress [52]. Previous studies showed that the effect of low temperature on antioxidant enzyme activities was closely related to the duration of the low-temperature stress, while the activity of antioxidant enzymes versus the duration of cold stress showed an initially increasing trend followed by a decrease [18]. In the experiments reported herein, three days of cold treatment significantly increased H_2_O_2_ content in rice seedlings. Concomitantly, POD, APX, and SOD activities significantly decreased, whereas CAT activity increased. Some studies have reported that SOD, APX, and POD activities were high in the first few hours of the initiation of the plant’s cold-stress response, but they decreased significantly with the extension of stress time [18,53]. Consistent with our findings, unlike SOD, CAT plays a more important role in the cold-stress response; short-term low-temperature treatment (6 days) was found to not reduce but increase CAT activity [18,53]. Further, under cold conditions, the exogenous application of BR significantly reduced H_2_O_2_ content in rice seedlings and significantly increased POD, APX, CAT, and SOD activities. This shows that exogenous application of BR can significantly increase the activities of antioxidant enzymes in rice seedlings under cold conditions, thereby improving the ability to scavenge and decompose H_2_O_2_, reduce H_2_O_2_ content, and ultimately enhance cold resistance of rice seedlings.

In addition to maintaining plant homeostasis through the antioxidant enzyme system, the non-enzymatic ROS scavenging system composed of ascorbic acid-glutathione is a major plant-resistance system to environmental stress [54,55]. GSH is primarily involved in ROS scavenging in two ways. The first consists of forming a redox cycle with AsA to consume H_2_O_2_, and the second is to indirectly scavenge ROS by regulating related antioxidant enzyme activities to participate in the enzymatic reactions for scavenging ROS [56]. Liu reported that BR treatment enhanced the AsA-GSH cycle in pepper leaves under cold stress by increasing the APX activity and AsA and GSH contents of alpine mustard calli cultured under cold stress [57]. In this experiment, three days of cold treatment significantly increased GSH, GSSG, and DHA contents, whereas the same treatment decreased AsA content and GSH/GSSG and AsA/DHA ratios in rice seedlings. However, exogenous application of BR under cold significantly increased GSH content, while reducing that of GSSG, and doubling the GSH/GSSG ratio. On the contrary, AsA, DHA, and AsA/DHA did not change significantly. This indicates that exogenous BR treatment under cold conditions increased GSH content and scavenge ROS by increasing antioxidant enzyme activity. The reduction of rice seedlings under cold conditions was alleviated, and the oxidation level was reduced to reduce cold-stress-induced peroxidative damage to rice seedlings.

### 4.4. BR Enhanced Cold-Resistance-Related Gene Expression to Enhance the Cold Resistance of Rice Seedlings

Previous studies have led to the conclusion that cold resistance of rice seedlings is controlled by several genes [15,16,17,58,59,60,61,62]. For example, *COLD1* encodes a protein that interacts with the α subunit RGA1 of G protein to sense cold conditions, and then activates Ca^2+^ channels to enhance cold tolerance in rice [15]. In turn, *OsMyb* is an MYB-like transcription factor containing a single DNA-binding repeat that not only mediates sucrose signaling in rice, but also plays a key role in rice adaptation to cold stress [58]. Additionally, *OsWRKY45* negatively regulates the cold tolerance of rice via crosstalk with abscisic acid (ABA)-signaling; rice with the *OsWRKY45* gene showed a lower survival percentage under cold-stress conditions than rice without it did [59]. In this study, BR might maintain a high level of Ca^2+^, sucrose, and ABA-signal transduction in cells to ensure timely physiological responses to maintain cellular homeostasis by increasing the relative expression of *COLD1* and *OsMyb* and reducing the relative expression of *OsWRKY45-1* under cold stress.

*TERF2* plays an important regulatory role in the abiotic stress response in rice. Overexpressed *TERF2* not only increases the content of osmotic substances and chlorophyll but also reduces ROS and malondialdehyde contents and electrolyte leakage [17]. *OsICE1* is a membrane-associated transcription factor that regulates the expression of cold-stress-induced upstream transcription-factor genes, such as *OsDREB1B* and *OsHsfA3*, thereby adapting rice to cold environments [60]. *OsFer1* is a light-regulated rice ferritin gene involved in resistance to iron-mediated oxidative stress and regulates cold tolerance in rice downstream of *TERF2* [61]. The transcription level of *OsSODB* directly affects Fe-SODs (SODB) contents, which play an important role in protection against oxygen toxicity [62]. BR treatment significantly increased the relative expression levels of all the above-mentioned genes under cold stress, indicating that BR improved osmotic substance contents and reduced the ROS burst to alleviate cold stress symptoms.

In rice, *OsLti6a* and *OsLti6b* are cold-sensitive genes, especially at the seedling stage, which can protect plasma membrane integrity under cold stress [16]. In this study, the expression of *OsLti6a* significantly increased after application of BR, suggesting that BR treatment may improve cytoplasmic membrane integrity in rice growing under cold stress to improve cold resistance.

**Figure 4:**
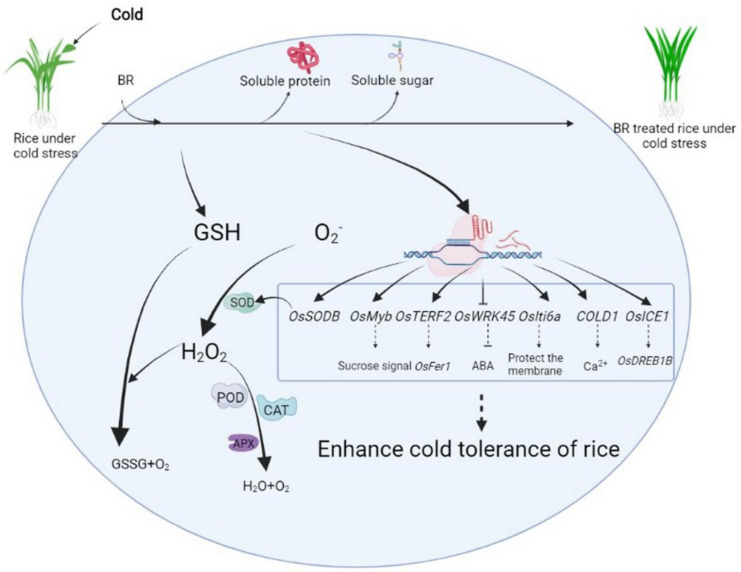
A model showing the alleviation of cold stress symptoms in rice by BR.

A model based on the results summarized above was prepared to illustrate the mechanisms of alleviation of cold stress in rice by BR (Figure 4). The addition of BR to the substrate significantly increased soluble sugar and protein contents to alleviate the cold-stress-induced osmotic stress. BR increased the activity of antioxidant enzymes, such as SOD, POD, CAT, and APX; this effect was accompanied by an increase in GSH content and the GSH/GSSG ratio, thereby alleviating cold-induced peroxidation damage. In addition, BR regulated the expression of genes related to sucrose signaling, Ca^2+^ signaling, the ABA signaling pathway, antioxidant synthesis, and membrane integrity, which are relevant to cold tolerance in rice, thereby improving the overall resistance to cold in rice seedlings.

## 5. Conclusions

BR increased soluble sugar and protein contents, antioxidant enzyme activity, GSH content, and the GSH/GSSG ratio, and regulated the expression of genes related to sucrose signaling, Ca^2+^ signaling, the ABA signaling pathway, antioxidant synthesis, and membrane integrity to improve cold resistance in rice seedlings. These results provide a theoretical basis for building cold tolerance in rice during early seedling development, and in breeding of cold-tolerant rice.

## Figures and Tables

**Table 1:** Treatment and control group settings.

	Treatments	Temperature/°C	
		12 h	12 h
W + H	Water	30	20
B + H	BR	30	20
W + L	Water	15	10
B + L	BR	15	10

Note: “W + H” represents the seedling substrate without BR application or cold treatment (control), “B + H” represents the seedling substrate with BR application but without cold treatment, “W + L” represents the seedling substrate without BR application but with cold treatment, and “B + L” represents the seedling substrate with BR and cold treatment.

**Table 2:** The effect of BR on germination parameters of rice seedlings exposed to contrasting temperature regimes.

Treatments	Germination Potential (%)	Germination Percentage (%)
W + H	84.33 ± 0.57b	90.33 ± 1.52b
B + H	90.33 ± 1.52a	97.33 ± 0.57a
W + L	79.66 ± 2.88c	87.66 ± 0.57c
B + L	83.66 ± 1.52b	89.33 ± 0.57bc

Note: “W + H” represents the seedling substrate without BR application or cold treatment (control), “B + H” represents the seedling substrate with BR application but without cold treatment, “W + L” represents the seedling substrate without BR application but with cold treatment, and “B + L” represents the seedling substrate with BR and cold treatment application. Different letters in the tables indicate that the differences between treatment means were significant (*p* < 0.05).

**Table 3:** The impact of BR on growth parameters of rice seedlings exposed to different temperatures.

Treatments	Plant Height (cm)	Plant Weight (g/100 Plants)	Leaf Age (d)	Root Number	Shoot Width (cm/10 Plants)
W + H	22.10 ± 0.33a	1.22 ± 0.02b	2.13 ± 0.08a	5.88 ± 0.32a	1.48 ± 0.02b
B + H	21.95 ± 0.64a	1.23 ± 0.03b	2.19 ± 0.04a	5.74 ± 0.25a	1.58 ± 0.02a
W + L	18.02 ± 0.15c	1.21 ± 0.02b	1.94 ± 0.00b	4.85 ± 0.20b	1.43 ± 0.02c
B + L	18.76 ± 0.05b	1.28 ± 0.01a	1.92 ± 0.01b	5.55 ± 0.04a	1.46 ± 0.01bc

Note: “W + H” represents the seedling substrate without BR application or cold treatment (control), “B + H” represents the seedling substrate with BR application but without cold treatment, “W + L” represents the seedling substrate without BR application but with cold treatment, and “B + L” represents the seedling substrate with BR and cold treatment application. Different letters in the tables indicate that the differences between treatment means were significant (*p* < 0.05).

**Table 4:** The effect of BR on H_2_O_2_ content and antioxidant enzyme activity in rice seedlings exposed to contrasting temperature regimes.

Treatments	H_2_O_2_ (μmol g^−^^1^ FW)	SOD (U g^−1^ FW)	POD (U g^−1^ FW)	APX (nmol min^−1^ g^−1^ FW)	CAT (nmol min^−1^ g^−1^ FW)
W + H	10.38 ± 0.33ab	654.42 ± 27.97b	4385.39 ± 24.50a	5333.04 ± 276.17a	332.37 ± 1.60c
B + H	8.18 ± 0.76c	637.73 ± 9.92b	3814.92 ± 52.51b	4272.29 ± 224.86b	342.25 ± 9.01bc
W + L	10.87 ± 0.35a	590.49 ± 13.84c	3756.26 ± 109.20b	3559.40 ± 11.59c	355.94 ± 2.31b
B + L	9.64 ± 0.84b	698.39 ± 11.16a	4157.98 ± 278.11a	4402.12 ± 311.14b	432.07 ± 13.41a

Note: “W + H” represents the seedling substrate without BR application or cold treatment (control), “B + H” represents the seedling substrate with BR application but without cold treatment, “W + L” represents the seedling substrate without BR application but with cold treatment, and “B + L” represents the seedling substrate with BR and cold treatment application. Different letters in the tables indicate that the differences between treatment means were significant (*p* < 0.05).

## Data Availability

The raw data supporting the conclusions of this manuscript will be made available by the authors, without undue reservation, to any qualified researcher.

## Supplementary Materials

The following supporting information can be downloaded at: https://www.mdpi.com/article/10.3390/life12060833/s1, Table S1: Growth parameters and germination rates of rice seedlings treated with different BR concentrations. Different letters in the tables indicate that the differences between treatment means were significant (*p* < 0.05). Table S2: The sequence of primers in rice. Refs. [15,16,17,58,59,60,61,63] are mentioned in Supplementary Materials.

## Abbreviations

Superoxide dismutase	SOD
Catalase	CAT
Peroxidase	POD
Ascorbate peroxidase	APX
Glutathione	GSH
Oxidized glutathione	GSSG
2-nitrobenzoic acid	DTNB
2-vinylpyridine	2-VP
Reactive oxygen species	ROS
