# Supplementary material for: Unearthing the Alleviatory Mechanisms of Brassinolide in Cold Stress in Rice
Source: Life (Basel). 2022 Jun 2;12(6):833. doi: 10.3390/life12060833 (PMC9225285; doi:10.3390/life12060833)
Supplement: Supplementary file 1 [file life-12-00833-s001.zip › life-1723109-supplementary.pdf]

Supplementary Table S1. Growth parameters and germination rates of rice seedlings treated with different BR concentrations. Different letters in the tables indicate that the differences between treatment means were significant ($p < 0.05$).

Treatment	Plant height (cm)	Leaf age (d)	Root number	Germination percentage (%)
W + H	21.58 ± 0.44ab	2.55 ± 0.00a	7.47 ± 0.42ab	85.33 ± 1.53b
0.0001% B + H	22.07 ± 0.66ab	2.59 ± 0.01a	8.00 ± 0.41ab	92.00 ± 1.00a
0.0005% B + H	22.13 ± 0.54a	2.63 ± 0.03a	7.90 ± 0.18ab	90.67 ± 1.15a
0.001% B + H	20.20 ± 0.66b	2.52 ± 0.03a	8.25 ± 0.34a	90.33 ± 1.53a
W + L	18.09 ± 0.59c	1.89 ± 0.05b	6.80 ± 0.23d	73.00 ± 1.00c
0.0001% B + L	20.56 ± 0.74b	2.07 ± 0.49b	7.50 ± 0.02b	92.66 ± 0.58a
0.0005% B + L	17.86 ± 0.63c	2.04 ± 0.29b	7.10 ± 0.02cd	83.33 ± 0.58b
0.001% B + L	17.95 ± 0.85c	2.00 ± 0.61b	7.45 ± 0.03b	83.00 ± 1.00b

Supplementary Table S2. Sequences of primers used in this study.

Gene	Forward sequence (5′-3′)	Reversed sequence (5′-3′)	References
COLD1	CAGGATATCAAAAGCTTGGATG	GCAGCTATCTTTGCTTGACG	[15]
OsICE1	ATGAGAATCTTCGGCAAGGA	CGTGGAACACCCTGGAGAT	[60]
TERF2	TTGTCATCCACCGACCTATG	TCACCATCTCCTCTCCTCC	[17]
OsLti6a	GCTGTCTGGGTCATCACCAAG	ACAGGGACGAGAAGGTGCAG	[16]
OsTrx23	AGAACACCATCGTGAAGCAC	CTATCTACAAGCTTGCCAGCAT	[17]
OsSODB	AGGCTGTTGCTGTGCTTGCT	GTAGGATTGAAGTGTGGTC	[17]
OsFer	GAGGAGACTGTGTGAAGGGC	ATATTGTTCCTTATTGGCTGCC	[60,61]
OsWRKY45	TTCCTTGTTGATGTGTCGTCTCA	CCCCCAGCTCATAATCAAGAAC	[59]
OsMyb	ATCGCCAAGAGCATTCCTG	AATCCGAGCAGAAGAAGGC	[58]
OsHistone	GGTCAACTTGTTGATTCCCCTCT	AACCGCAAAATCCAAAGAACG	[63]
